# Adaptation of a RAS pathway activation signature from FF to FFPE tissues in colorectal cancer

**DOI:** 10.1186/s12920-016-0225-2

**Published:** 2016-10-19

**Authors:** Bernard Omolo, Mingli Yang, Fang Yin Lo, Michael J. Schell, Sharon Austin, Kellie Howard, Anup Madan, Timothy J. Yeatman

**Affiliations:** 1 Division of Mathematics and Computer Science, University of South Carolina-Upstate, 800 University Way, Spartanburg, SC 29303 USA; 2 Gibbs Cancer Center and Research Institute, 101 E Wood Street, Spartanburg, SC 29303 USA; 3 Genomic Services, Covance Genomics Lab, 9911 Willows Road, Suite 175, Redmond, WA 98052 USA; 4 Department of Biostatistics and Bioinformatics, H. Lee Moffitt Cancer Center and Research Institute, 12902 Magnolia Drive, Tampa, FL 33612 USA

**Keywords:** Colorectal cancer, FF (fresh-frozen), FFPE (formalin-fixed, Paraffin embedded), Microarray, NanoString, RAS pathway signature, RNASeq

## Abstract

**Background:**

The *KRAS* gene is mutated in about 40 % of colorectal cancer (CRC) cases, which has been clinically validated as a predictive mutational marker of intrinsic resistance to anti-EGFR inhibitor (EGFRi) therapy. Since nearly 60 % of patients with a wild type *KRAS* fail to respond to EGFRi combination therapies, there is a need to develop more reliable molecular signatures to better predict response. Here we address the challenge of adapting a gene expression signature predictive of RAS pathway activation, created using fresh frozen (FF) tissues, for use with more widely available formalin fixed paraffin-embedded (FFPE) tissues.

**Methods:**

In this study, we evaluated the translation of an 18-gene RAS pathway signature score from FF to FFPE in 54 CRC cases, using a head-to-head comparison of five technology platforms. FFPE-based technologies included the Affymetrix GeneChip (Affy), NanoString nCounter™ (NanoS), Illumina whole genome RNASeq (RNA-Acc), Illumina targeted RNASeq (t-RNA), and Illumina stranded Total RNA-rRNA-depletion (rRNA).

**Results:**

Using Affy_FF as the “gold” standard, initial analysis of the 18-gene RAS scores on all 54 samples shows varying pairwise Spearman correlations, with (1) Affy_FFPE (*r =* 0.233, *p* = 0.090); (2) NanoS_FFPE (*r =* 0.608, *p* < 0.0001); (3) RNA-Acc_FFPE (*r =* 0.175, *p* = 0.21); (4) t-RNA_FFPE (*r =* −0.237, *p* = 0.085); (5) and t-RNA (*r =* −0.012, *p* = 0.93). These results suggest that only NanoString has successful FF to FFPE translation. The subsequent removal of identified “problematic” samples (*n =* 15) and genes (*n =* 2) further improves the correlations of Affy_FF with three of the five technologies: Affy_FFPE (*r =* 0.672, *p* < 0.0001); NanoS_FFPE (*r =* 0.738, *p* < 0.0001); and RNA-Acc_FFPE (*r =* 0.483, *p* = 0.002).

**Conclusions:**

Of the five technology platforms tested, NanoString technology provides a more faithful translation of the RAS pathway gene expression signature from FF to FFPE than the Affymetrix GeneChip and multiple RNASeq technologies. Moreover, NanoString was the most forgiving technology in the analysis of samples with presumably poor RNA quality. Using this approach, the RAS signature score may now be reasonably applied to FFPE clinical samples.

**Electronic supplementary material:**

The online version of this article (doi:10.1186/s12920-016-0225-2) contains supplementary material, which is available to authorized users.

## Background

Colorectal cancer (CRC) is the third most common cancer in men and women [1]. Nearly one-third of the patients will eventually die of the disease. Hyperactivation of the RAS signaling pathway is a driver of many cancers, including CRC [2, 3]. Activating mutations in the K-ras proto-oncogene (*KRAS*) are found in approximately 40 % of colorectal tumors [4]. Thus, the RAS pathway activation has become a major focus of drug targeting efforts, including prediction of response to targeted therapies [5–8]. For example, the epidermal growth factor receptor (EGFR) is a major therapeutic target in metastatic colorectal cancer [7–14]. The fact that nearly 60 % of patients with a wild type *KRAS* fail to respond to combination therapies involving EGFRi [5, 15], however, strongly suggests that there are additional genes, beyond *KRAS*, that contribute to RAS pathway activation. It has been recently reported that mutations in *BRAF* and *NRAS* that also activate the RAS pathway may account for EGFRi therapy resistance in some of the wild-type *KRAS* CRCs [7, 10, 12, 14].

A number of gene expression signatures have been developed using multiple types of cancer cell lines and human fresh frozen (FF) samples to predict RAS pathway dependence in association with drug response [2, 3]. For example, a 147-gene RAS pathway signature has been reported to be superior to *KRAS* mutation status alone for the prediction of dependence on RAS signaling, and it could predict response to PI3K and RAS pathway inhibitors in lung and breast tumors [3]. Low RAS pathway signature score was associated with a higher cetuximab response rates in a retrospective analysis of metastatic CRC [3]. Another RAS pathway signature (18 genes) was developed from multiple types of cancer cell lines and human tumors, including CRC, to specifically assess MEK functional output and activation of the RAS/RAF/MEK/ERK pathway [2]. While measuring mutations in individual genes such as *KRAS* and *NRAS* can predict EGFRi response, their level of accuracy is low with up to 60 % of patients still not-responding [15]. For this reason, multi-gene expression signatures hold promise in being able to more robustly assess pathway activation than single gene mutations, and thus there is an interest in translating them for use with FFPE clinical samples.

One of the challenges in using these gene expression signature scores is that many have been developed using fresh-frozen (FF) tissues on the Affymetrix GeneChip (microarray) platform. In order for these signature scores to be clinically useful, they need to be adapted to the more commonly available archival formalin-fixed paraffin-embedded (FFPE) tissues [16, 17]. However, microarrays that can assess thousands of transcripts are not only expensive but also lack reproducibility, especially when evaluating FFPE samples having low RNA quality [18, 19]. Determinants of RNA quality from FFPE samples have been reported to include storage time and conditions, fixation time and specimen size [20]. RT-qPCR and NanoString technologies have been reported to be useful for gene expression quantification in FFPE tissues [17, 21–23]. However, the recently developed, probe-based NanoString method was shown to be superior to the RT-qPCR approach in archived FFPE samples [22].

To date, the RAS pathway signatures developed in FF samples for prediction of drug response have not been validated in CRC using FFPE samples. Thus, in this study, we elected to evaluate the translation of an 18-gene RAS signature score [2] from FF to FFPE in 54 selected CRC cases in a head-to-head comparison of five technology platforms: Affymetrix GeneChip (Affy), NanoString nCounter™ (NanoS), whole genome RNASeq (Illumina RNA-Access (RNA-Acc), targeted RNASeq (t-RNA), and Illumina Total stranded RNA-rRNA-depletion (rRNA).

## Methods

### Tissue sample selection

Fifty-four (54) FFPE evaluable tumor specimens were selected from a larger multi-center cohort of 468 well-characterized colorectal adenocarcinoma patients whose tissues were obtained between October 2006 and September 2010 [24]. In all cases, tissue and clinical data were collected on patients with the University of South Florida institutional review board approval [25]. All tumors were collected from curative survival resections and snap frozen in liquid nitrogen within 15–20 min of extirpation. The sample cohort was composed of tumor samples that were available as matched fresh-frozen (FF) and formalin-fixed paraffin-embedded (FFPE) pairs. As shown in Additional file 1, the 54 samples had mutant *KRAS* (25/54 or 46 %) and *BRAF* (2/54 or 4 %), but no *NRAS* mutations.

The Affymetrix GeneChip, NanoString, whole genome RNASeq, and targeted RNASeq assays on the 54 FFPE samples were performed at LabCorp, Inc., Seattle, USA. Whole genome RNASeq was further comprised of two library preparation methods: Illumina RNA-Access (RNA-Acc) and Illumina Total stranded RNA-rRNA-depletion (rRNA), which were analyzed as separate datasets. Targeted RNA sequencing data (t-RNA) was based on the RAS 18-gene signature [2].

**Fig. 1:**
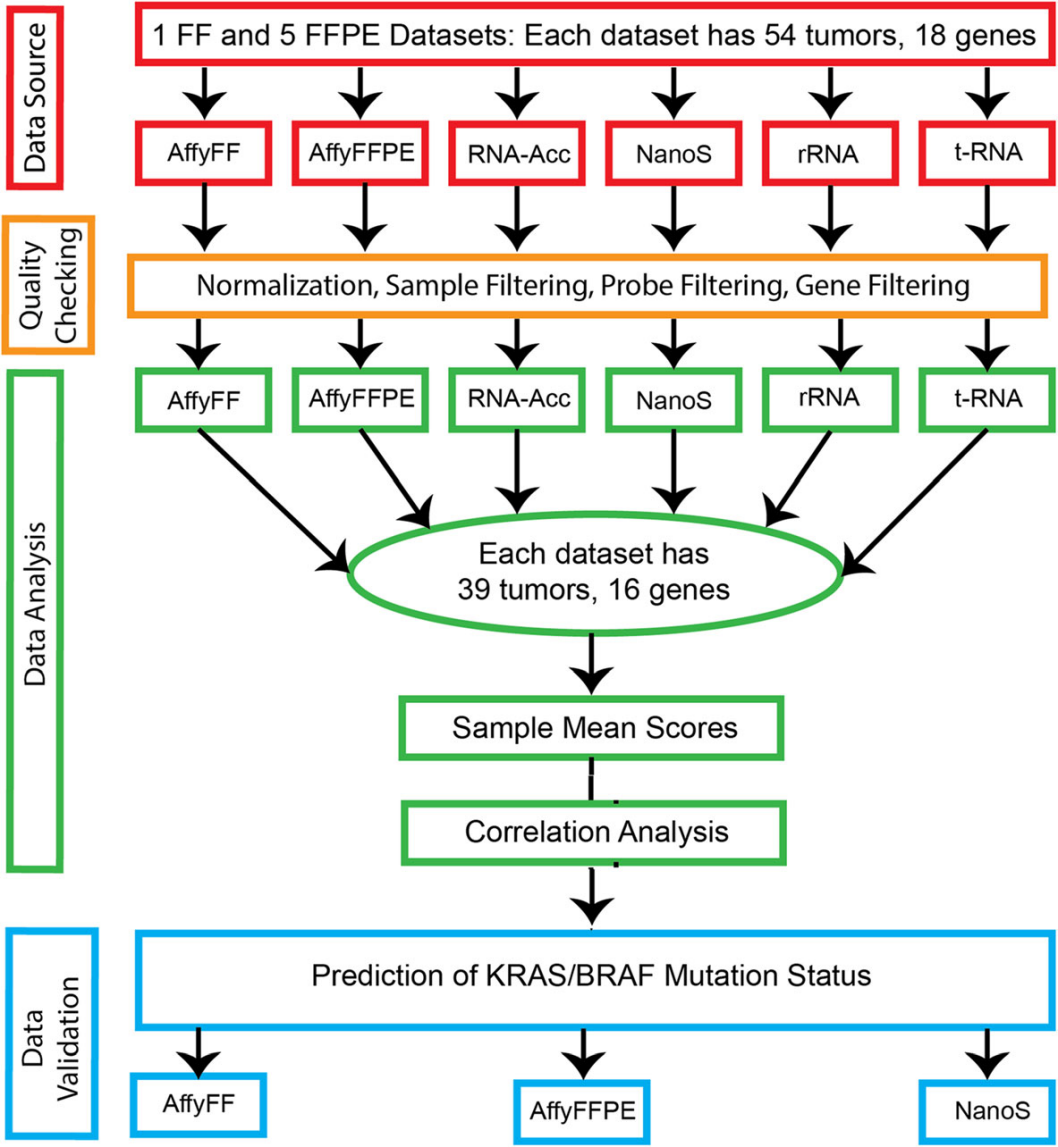
Flow-chart of the procedure followed in the pre-processing and analysis of the data. Six datasets (1 FF and 5 FFPE, each with 54 samples and 18 genes) underwent quality control procedures before analysis. Thirty-nine [39] “good” samples and 16 “good” genes were retained. Correlation analyses were performed using mean scores from the sample pairs. The predictive ability of the 16–gene set was validated using the Affymetrix FF, Affymetrix FFPE and NanoString gene expression data, by the PAM method

The flowchart (see Fig. 1) below shows the steps followed in the pre-processing and analysis of the data. The statistical methods used include [1] the Robust Microarray Average (RMA) method [26] for the normalization of Affy_FF and Affy_FFPE samples; [2] principal component analysis (PCA) [19, 27] to identify “bad” samples from the Affy_FFPE data; [3] correlation analyses among the datasets; and [4] the nearest shrunken centroids algorithm for predicting the mutation type of a sample.

### Data pre-processing

Both the 54 Affy_FF and matching Affy_FFPE samples were normalized using the RMA method [26]. For the NanoString data, we used the reference (housekeeping) gene normalization method, as described in the nCounter® Expression Data Analysis Guide (available at http://www.nanostring.com/media/pdf/MAN_nCounter_Gene_Expression_Data_Analysis_Guidelines.pdf). The 11 housekeeping genes were *BIRC6, EMC8, HADHA, MAEA, MRPL18, ORMDL1, PSMD11, RBM4, STX6, TRIM39*, and *UBE2K*. The geometric mean of these reference genes was obtained for each sample (lane) and the average of these means across all samples calculated. The normalization factor for each sample was the overall mean divided by the geometric mean. We multiplied this factor by the mRNA transcript count for each of the 18 RAS genes in the sample. For the targeted RNA data, we used median normalization. For that platform, we obtained the median for each of the samples and subtracted this number from each of the gene counts for the sample. Notably, global normalization using median-centering is commonly used to correct for sample-specific bias (due to experimental artefacts) and render the gene expression levels comparable in differential gene expression analysis in microarrays [28]. With the advent of RNASeq technology, the method has been adopted to render counts from different samples, which may have been sequenced to different depths, comparable [29]. Thus, gene expression values could be positive or negative numbers relative to a reference (e.g. median). For the whole transcriptome RNASeq (RNA-Acc and rRNA) platforms, the data was first processed by STAR aligner [30] and cufflinks [31], then the resulting FPKM was log2-transformed and z-score-normalized.

### Probe filtration

After normalization, filtration of probes was performed for both the Affy_FF and Affy_FFPE data. Probes were retained if they had at least 1.5-fold change in either direction of the median expression level in at least 20 % of the samples and if they had at most 50 % missing values across the samples. The entire probe filtration process was implemented by the BRB-ArrayTools software [32]. The NanoS_FFPE, RNA-Acc_FFPE, t-RNA and rRNA_FFPE datasets did not have probe-level data and so were not subjected to the probe filtration process.

### Calculation of RAS pathway activation scores before probe filtration

The next step was to calculate the RAS pathway activation scores from the normalized 54 Affy_FF and Affy_FFPE samples. For genes with multiple probes/probesets in the dataset, probes/probesets from the same gene were averaged to yield one value of the expression level for each gene. The mean of these expression levels across the 18 RAS genes was calculated for each sample to yield the sample mean score. The sample mean scores for the NanoS_FFPE, RNA-Acc_FFPE, t-RNA_FFPE and rRNA_FFPE were obtained by averaging across the 18 RAS genes.

### Calculation of RAS pathway activation scores after probe filtration

The probe filtration resulted in a reduction of probes from 60,607 to 23,765. Some genes that were represented by only one probe in the dataset were filtered out in this filtration process. For the remaining genes with multiple probes/probesets, the probes/probesets were reduced to one per gene by selecting the probe with the highest mean signal strength across the samples. The mean expression levels across the remaining 16 RAS genes were calculated for each sample to yield the sample mean score. The sample mean scores for the NanoS_FFPE, RNA-Acc_FFPE, t-RNA_FFPE and rRNA_FFPE were obtained by averaging across the 16 RAS genes.

### Statistical analysis

The FF - FFPE sample pairs of mean scores were used in the correlation analyses, using SAS software version 9.4 (SAS Institute, Cary, NC, USA). There were 15 possible combinations of the sample pairs, among the six datasets (Affy_FF, Affy_FFPE, NanoS_FFPE, RNA-Acc_FFPE, t-RNA-FFPE, and rRNA_FFPE), yielding 15 pairwise Spearman correlations. We also assessed the effect of removing “bad” samples and probes on the Spearman correlations across the five platforms. To identify the “bad” samples, we performed a principal component analysis (PCA) of the 54 Affy_FFPE samples, with the entire set of 60,607 probes, to generate the first two principal components (PC1 and PC2), using the SAS software version 9.4. The PC1 and PC2 scores were identified as the eigenvectors of the covariance matrix of the 54 Affy_FFPE samples that accounted for the highest and the second-highest variation in the data, respectively. A scatterplot of PC2 vs PC1 was used to show the location of the possibly “bad” samples.

Samples were classified as either *KRAS*/*BRAF* mutant or *KRAS*/*BRAF* wild-type (WT). The nearest shrunken centroids algorithm [33] was employed in predicting the mutation type of a sample, based on the gene expression profiles of the 16 genes from the 18-gene RAS signature. This algorithm was implemented by the Prediction Analysis of Microarrays (PAM) tool in BRB-ArrayTools software [32]. The algorithm builds several linear models (classifiers) containing up to 16 genes and selects the model with the minimal prediction error. The prediction error of the models are estimated using leave-one-out cross-validation (LOOCV) as described in [34]. For each leave-one-out training set, the entire model building process was repeated, including the gene selection process. The proportion of times when classifiers incorrectly predicted the class (miss-classification rate) of the excluded samples was recorded for the entire training set of samples.

## Results

### NanoString effectively translates the 18 gene RAS scores from FF to FFPE in all 54 samples

A gene expression RAS pathway signature [2], comprised of 18 genes (*DUSP4, DUSP6, ELF1, ETV4, ETV5, FXYD5, KANK1, LGALS3, LZTS1, MAP2K3, PHLDA1, PROS1, S100A6, SERPINB1, SLCO4A, SPRY2, TRIB2* and *ZFP106)*, was used to evaluate FF to FFPE translation on the 54 samples (see Table 1A). Results show the pairwise Spearman correlations of Affy_FF scores (“gold” standard) with five sets of scores obtained from [1] Affy_FFPE; [2] NanoS_FFPE; [3] RNA-Acc_FFPE; [4] t-RNA_FFPE; and [5] rRNA_FFPE. Only NanoS_FFPE appeared to have successful FF to FFPE translation for the 18-gene RAS scores (*r =* 0.608, *p* < 0.0001) when all samples were utilized. Notably, among the five FFPE technology platforms, pairwise correlations between Affy_FFPE and each of NanoS_FFPE, RNA-Acc_FFPE and t-RNA_FFPE were significant.

**Table 1:** Spearman correlations for the 18-gene RAS signature scores among six datasets, including Affy_FF, NanoS_FFPE, RNA-Acc_FFPE, t-RNA_FFPE, and rRNA_ FFPE on 54 and 39 samples

Mean Score	Affy FF	Affy FFPE	NanoS FFPE	RNA-Acc FFPE	t-RNA FFPE	rRNA FFPE
A. 54 samples						
Affy FF	1	0.233 (0.090)	0.608 (<0.0001)	0.175 (0.207)	−0.237 (0.085)	−0.012 (0.934)
Affy FFPE		1	0.399 (0.003)	0.760 (<0.0001)	0.278 (0.042)	0.260 (0.058)
NanoS FFPE			1	0.473 (0.0003)	−0.207 (0.134)	0.033 (0.814)
RNA-Acc FFPE				1	0.262 (0.056)	0.225 (0.102)
t-RNA FFPE					1	0.142 (0.306)
rRNA FFPE						1
B. 39 samples						
Affy FF	1	0.556 (0.0002)	0.631 (<0.0001)	0.261 (0.109)	−0.287 (0.076)	0.123 (0.455)
Affy FFPE		1	0.832 (<0.0001)	0.778 (<0.0001)	0.006 (0.973)	0.091 (0.581)
NanoS FFPE			1	0.733 (<0.0001)	−0.177 (0.282)	0.099 (0.551)
RNA-Acc FFPE				1	0.043 (0.797)	0.090 (0.587)
t-RNA FFPE					1	−0.071 (0.668)
rRNA FFPE						1

### Identification and removal of 15 “poor” quality samples improves the FF - FFPE correlations

**Fig. 2:**
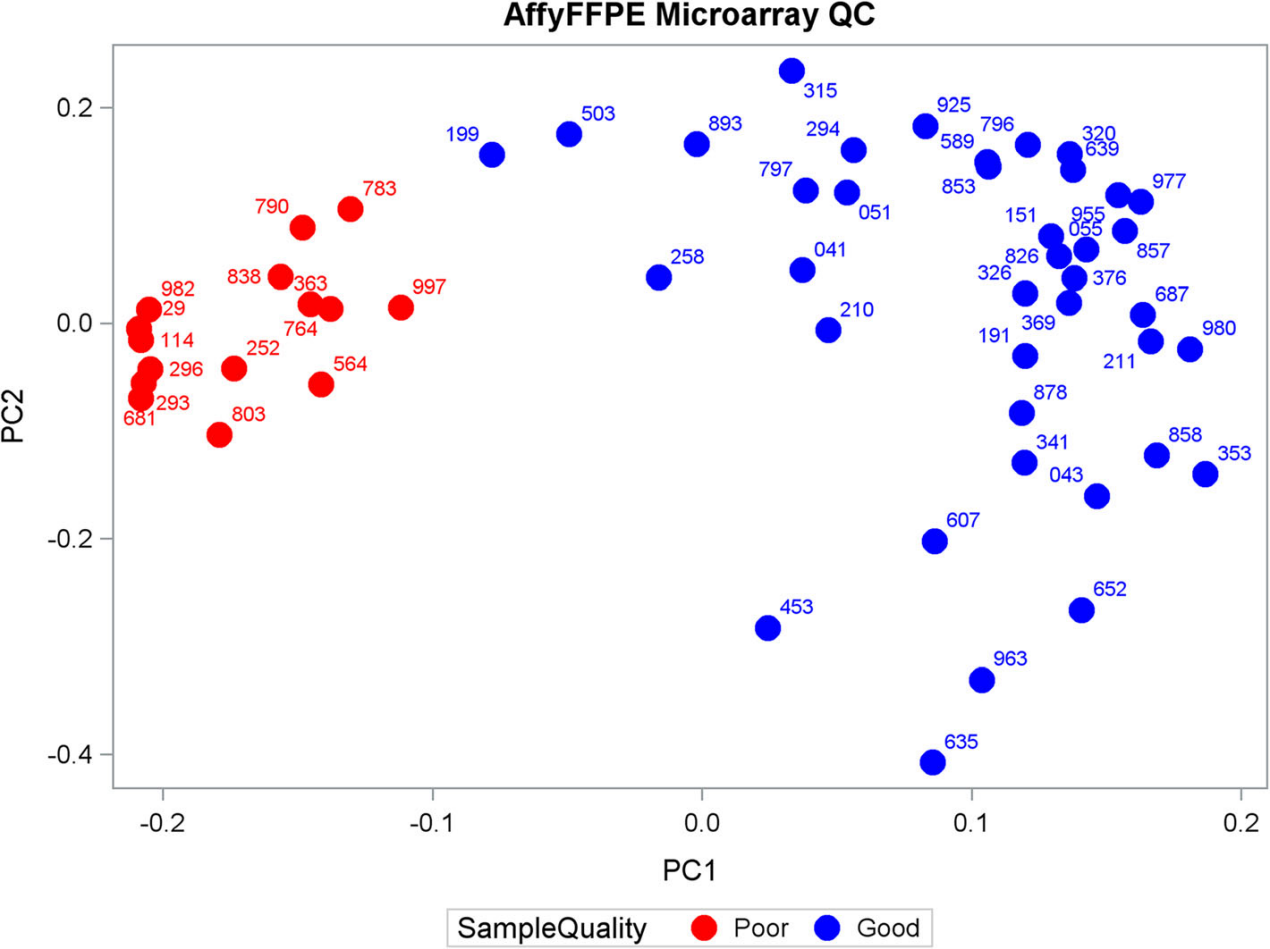
Scatterplot of the second vs. first principal component (PC2 vs PC1) for the 54 Affymetrix FFPE samples. The 15 “bad” samples (with low PC1 scores) are colored red and were excluded from subsequent analyses. Each sample was labeled using the last 3 digits of its name (barcode)

**Fig. 3:**
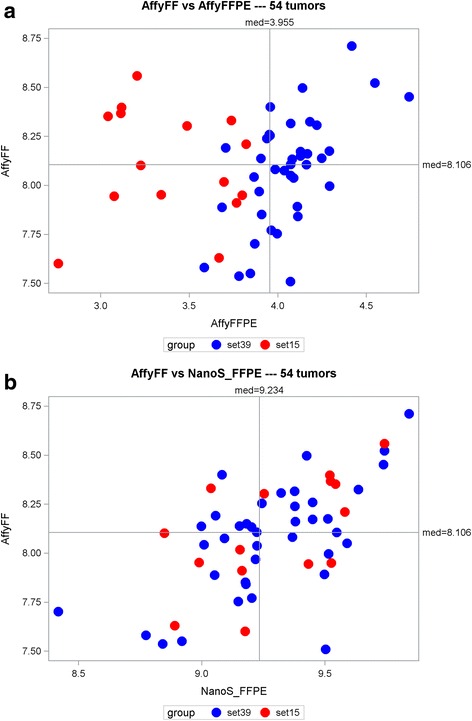
Scatterplots of the Affymetrix FF vs. Affymetrix FFPE (**a**) and NanoString FFPE (**b**) mean scores for the 54 samples. The red circles represent the 15 samples with “poor” RNA quality

Sfakianos et al. used the PCA procedure to detect outliers and showed that the outliers were associated with poor quality samples [19]. More recently, Guinney et al. performed quality control analysis for outlier detection using PCA [11]. We adopted this procedure to identify samples that could possibly have “poor” RNA quality. A scatterplot (see Fig. 2) of the first and second principal component (PC1 and PC2) scores identified fifteen samples with low PC1 scores (hereby less than - 0.10) that were considered to be “outliers”, or samples likely to have “poor” RNA quality. Notably, as compared to other 39 “good” samples (see Table 1B), most of 15 samples identified also had low standard deviations (signal-to-noise ratios) across probes (data not shown). Furthermore, these 15 samples all had below average Affy_FFPE mean scores in contrast to their wide-spread pattern for Affy-FF scores (see Fig. 3a). Thus, these data support the notion that low PC1 scores were likely associated with “poor” RNA quality, However, these 15 “bad” samples did not stand out among the NanoS_FFPE mean scores (see Fig. 3b), suggesting that NanoString technology may be more forgiving of poor RNA quality inherent to these samples. The 15 samples identified with potential “poor” quality were removed, leaving 39 samples available for subsequent analyses.

As shown in Table 1B, the removal of 15 “outlier” samples resulted in increases in three pairwise correlations among Affy_FF vs. [1] Affy_FFPE, [2] NanoS_FFPE and [3] RNA-Acc_FFPE. Notably, the correlation between Affy_FF and Affy_FFPE changed from being insignificant (*r =* 0.233, *p* = 0.090) to significant (*r =* 0.556, *p* = 0.0002).

Notably, while the 39 “good” samples had mutant *KRAS* (19/39) and *BRAF* (1/39), the 15 “bad” samples had mutant *KRAS* (6/15) and *BRAF* (1/15). No signification association was seen between sample RNA quality and *KRAS/BRAF* genotypes. For example, the Fisher’s exact test of association of *BRAF* mutation status and sample RNA quality was insignificant (*p* = 0.5711). This suggests that the “badness” of the 15 samples is likely not due to a biological reason (e.g. *BRAF* V600E enrichment), but rather﻿ to a “technical aspect of the sample preparation”.

### Reduction of probes and associated genes in attempt to improve further the FF-FFPE correlations

The 18 RAS signature genes were represented by 51 probes and 50 probesets, in the Affy_FF and Affy_FFPE datasets, respectively, with 48 probes in common to both. Probe selection was performed to exclude probes that were not sufficiently differentially-expressed across the 39 samples. The selection was performed from the entire set of 60,607 probes on 39 Affy_FFPE samples. We first filtered out those probes with less than 1.5-fold change in either direction of the probe’s median value and then filtered out those with at least 1.5 fold change but in less than 20 % of the samples, resulting in 23,765 probes in 10,031 genes. We then reduced the number of probes to one per gene, by selecting the probes with the highest mean expression values. Due to the probe selection, all probes from the genes *LZTS1* and *ZFP106* were dropped, yielding a 16-gene signature that was then applied to the 54 and 39 samples, respectively.

Removal of 2 “problematic” probes/genes, while retaining all 54 tumors, resulted in a modest increase in two pairwise correlations among Affy_FF vs. [1] Affy_FFPE and [2] NanoS_FFPE (comparing Table 1A vs. Table 2A). Of interest, the performance of t-RNA_FFPE and rRNA_FFPE were also improved with probe reduction.

**Table 2:** Spearman correlations for the 16-gene RAS signature scores among six datasets, including Affy_FF, NanoS_FFPE, RNA-Acc_FFPE, rRNA_FFPE, and t-RNA_ FFPE on 54 and 39 samples

Mean Score	AffyFF	Affy FFPE	NanoS FFPE	RNA-Acc FFPE	t-RNA FFPE	rRNA FFPE
A. 54 samples						
Affy FF	1	0.300 (0.028)	0.707 (<0.0001)	0.264 (0.054)	−0.195 (0.157)	0.019 (0.890)
Affy FFPE		1	0.361 (0.007)	0.773 (<0.0001)	0.296 (0.030)	0.369 (0.006)
NanoS FFPE			1	0.487 (0.0002)	−0.155 (0.264)	0.039 (0.779)
RNA-Acc FFPE				1	0.262 (0.056)	0.295 (0.031)
t-RNA FFPE					1	0.194 (0.159)
rRNA FFPE						1
B. 39 samples						
Affy FF	1	0.672 (<0.0001)	0.738 (<0.0001)	0.483 (0.002)	−0.228 (0.163)	0.174 (0.290)
Affy FFPE		1	0.845 (<0.0001)	0.754 (<0.0001)	0.014 (0.934)	0.170 (0.301)
NanoS FFPE			1	0.802 (<0.0001)	−0.096 (0.560)	0.150 (0.361)
RNA-Acc FFPE				1	0.053 (0.750)	0.182 (0.269)
t-RNA FFPE					1	−0.018 (0.915)
rRNA FFPE						1

Building to improve the model, the removal of both 15 samples and 2 probes/genes further enhanced the correlations of Affy_FF vs. Affy_FFPE, NanoS_FFPE, and RNA-ACC_FFPE (comparing Table 1A vs. Table 2B).

### Using a PAM classifier to predict *KRAS*/*BRAF* mutation status using the 16-gene expression data

The Affy_FF gene expression score might be considered a new “gold” standard because of its potential capacity to more inclusively identify tumors with RAS pathway activation not necessarily linked to RAS mutation. RAS mutation status, however, taken by itself, could also be considered a “gold” standard, and in fact is the current clinical standard used to qualify the administration of EGRFi therapies. We therefore sought to validate the known mutational status of previously sequenced CRC samples (*n =* 54) using our Affy_FF, Affy_FFPE and NanoS_FFPE datasets in conjunction with the modified 16-gene RAS signature score. In this regard, the samples were classified as either *KRAS*/*BRAF* mutant or *KRAS*/*BRAF* wild-type, resulting in two classes. Notably, no *NRAS* mutation was detected in the 54 samples (Additional File 1). For each dataset, we developed linear models utilizing gene expression profiles of the 16 genes to predict the class (mutation type) of future samples. Table 3 shows the sensitivity and specificity values for the classifier, together with the LOOCV miss-classification rates. Class prediction was performed using the gene expression data (*n =* 54) from the Affy_FF, Affy_FFPE and NanoS_FFPE samples. The 16-gene Affy_FF classifiers performed best in predicting *KRAS*/*BRAF* mutation status (error rate = 19 %), with an optimal sensitivity of 0.852 and specificity of 0.778. Reduction in sample size was ineffective in improving *KRAS*/*BRAF* mutation status predictions (results not shown). Table 4 shows the reduced gene sets in the selected predictive model (one with the minimal error of prediction) for each of the validation datasets (Affy_FF, NanoS_FFPE and Affy_FFPE) out of the 16 genes.

**Table 3:** Performance of the 16-gene PAM classifier on the 54 samples

Validation dataset	Class	Sensitivity^a^	Specificity^b^	LOOCV error rate
Affy FF	Mut	0.852 = 23/27	0.778 = 21/27	19 %
NanoS_FFPE	Mut	0.704 = 19/27	0.741 = 20/27	28 %
Affy_FFPE	Mut	0.519 = 14/27	0.889 = 24/27	30 %

*Note*: Samples were classified as either *KRAS*/*BRAF* mutant (Mut, *n =* 27) or *KRAS*/*BRAF* wild-type (WT, *n =* 27). Class prediction was performed using the Affy_FF, Affy_FFPE and NanoS_FFPE samples sets

^a^ = number of predicted mutants divided by number of true mutants

^b^ = number of predicted WT divided by number of true WT

**Table 4:** Genes in the predictive models of the 54-tissue PAM analyses

Validation dataset	Genes						
Affy_FF	*DUSP4*	*DUSP6*	*ETV4*	*ETV5*	*PHLDA1*	*SERPINB1*	*TRIB2*
NanoS_FFPE	*DUSP4*	*DUSP6*		*ETV5*		*SERPINB1*	
Affy_FFPE	*DUSP4*			*ETV5*			

*Note*: *DUSP4* and *ETV5* are the most common genes in the predictive models

## Discussion

Gene expression signatures have been identified for prediction of RAS pathway dependence and drug response [2, 3]. One obstacle to clinical translation is that these signatures were developed using cell lines and fresh frozen (FF) tissues, whereas usually only formalin-fixed, paraffin embedded (FFPE) tissue of lower quality is readily available for clinical use [19–22]. A number of studies have been reported on gene expression quantitation in FFPE samples using FF as a standard, usually employing one or two technologies, including RT-qPCR, NanoString, and/or Affymetrix GeneChip [19, 21, 22, 35–37]. In this study, we simultaneously compared five technology platforms: [1] Affymetrix GeneChip; [2] NanoString; [3] Illumina whole genome RNASeq RNA-Access; [4] Illumina Total RNA-stranded rRNA-depletion; and [5] targeted RNASeq. Analyses of 54 CRC samples were performed in a head-to-head comparison to identify the optimal method(s) for translating the RAS signature score [2] to FFPE tissues. For this purpose, we chose to calculate and compare individual tumor composite (multi-gene) scores rather than compare gene-level measurements in order to derive a more robust comparison of available technology platforms. Here, we found that while NanoString technology is the most forgiving in the analysis of samples with poor RNA quality, Affymetrix and RNA-Access may have potential for FF to FFPE translation upon removal of “outlier” samples.

The poor quality of RNA extracted from FFPE samples is thought to result from fixing procedures that cause RNA cross-linking and from RNA degradation over time in FFPE blocks depending on storage temperature [20, 21, 35–38]. While Lebbe and co-workers used the expression levels of a set of reference genes to construct a statistic for differentiating “bad” melanoma samples from “good” ones [36], Sfakianos et al. used PCA analysis to identify “bad” samples in ovarian cancer FFPE samples [19]. We adopted the PCA method to identify and filter out 15 “outlier” samples with “poor” RNA quality. The removal of the “outlier” samples improved the correlations of Affy_FF (as a “gold” standard) significantly with Affy_FFPE, but only slightly with NanoS_FFPE; the 15 “outlier” samples identified for the Affy_FFPE platform did not appear to be outliers for NanoS_FFPE. A plausible explanation is that in contrast to Affymetrix and RNASeq technologies, NanoString is a more “direct” technology (hybridization-based) to detect the number of RNA transcripts, so it does not need steps of mRNA reverse-transcription into cDNA and subsequent cDNA amplification. Reverse-transcription and cDNA amplification are known to be sensitive to the RNA quality issue caused by RNA cross-linking in FFPE samples.

Since multiple different gene-specific probes (used in Affymetrix technologies) may have different sensitivities to the RNA quality of FFPE samples [21], we used the mean signal scores for the probes coupled with their fold-change information, to filter out 2 genes that were insufficiently expressed across the 39 samples. Notably, our probe filtration approach here differs from the filtration methods used previously in the literature [39]. The removal of these two genes improved the FF to FFPE translation by both Affymetrix and NanoString methods. This indicates that the RNA quality and probe problems are two different confounding factors for the translation of the RAS signature scores. Notably, we observed poor correlations and no significant improvement upon removal of the “outlier” samples and/or “bad” probes for Illumina Total RNA-stranded rRNA-depletion, and targeted RNASeq. However, the cause was not clear.

Moreover, NanoString mean scores were most significantly and consistently correlated with Affymetrix FF, Affymetrix FFPE and RNA-Access mean scores, in the presence or absence of bad samples and probes. Furthermore, while our data suggest that removing “bad” samples can improve the translation of a test from FF to FFPE tissues in Affymetrix FFPE and RNA-Acces platforms, identifying samples with poor RNA quality is not always an easy and practical task. Within a potential future diagnostic setting, it is impractical to perform a PCA across multiple samples to identify “bad” samples. Even if this were practical, it is far from ideal to exclude patients from diagnostic assessment because their FFPE samples happened to have lower quality RNA than usual. In addition, attempting to identify and remove poor quality samples adds an additional step to any analysis. Thus, due to its lower apparent sensitivity to the RNA quality, NanoString technology is more practically useful in translation from FF to FFPE than are the Affymetrix and RNASeq technologies.

In the assessment of the predictive ability for *KRAS*/*BRAF* mutation status, the Affymetrix _FF 16-gene classifier produced the lowest misclassification rate (19 %) on the 54 samples. Our PAM analysis could further reduce the modified RAS pathway signature gene set from 16 to 7 genes in the Affy_FF classifier. Whereas all 18 genes were selected for capacity to identify MEK pathway activity independent of tumor genotype, the majority of the selected genes have particularly strong and direct relationships to the RAS/MEK/ERK pathway activation. *DUSP4/6* [40] and *PHLDA1* (*TGAD51*) [41] are known transcriptional targets of MEK/ERK. *ETV4/5* [42] can replace RAS/MAPK pathway activation and *TRIB2* can enhance ERK phosphorylation [43]. These relationships point to the strength of the signature genes identified by the algorithms applied to our sample sets. Of interest, *SERPINB1* was retained in Affy_FF and NanoS_FFPE sample sets but appeared to have no direct relationship to RAS pathway activation.

## Conclusions

Of the five technology platforms tested, NanoString technology was more adaptive to the translation of the RAS pathway signature from FF tissues to commonly available FFPE tissues than were the Affymetrix GeneChip and RNASeq technologies. NanoString was the most forgiving FFPE technology in reproducing the “gold” standard analysis on matched FF tissues. NanoString technology appears to rescue samples with poor RNA quality, permitting more samples to be scored. These critical analyses pave the way for a RAS pathway signature score to be used to assess FFPE CRC samples for applications such as prediction of EGFRi response to therapy.

## Additional file

Additional file 1: The 18 gene RAS scores, and KRAS/BRAF/NRAS mutation status for 54 CRC samples (15 “bad” samples are in bold; PC1: the first principal component; PC2: the second principal component; 1 = mutation present, 0 = mutation absent; * = score based on 16 genes) (XLSX 15 kb)

## Abbreviations

AffyFF: Affymetrix FF
AffyFFPE: Affymetrix FFPE
CRC: Colorectal cancer
FF: Fresh-frozen
FFPE: Formalin-fixed paraffin-embedded
IRON: Iterative rank-order normalization
LOOCV: Leave-one-out cross-validation
NanoS: NanoString
NPV: Negative predictive value
PAM: Prediction analysis of microarrays
PCA: Principal component analysis
PPV: Positive predictive value
RMA: Robust microarray average
RNA-Acc: RNA-Access
rRNA: rRNA-depletion
t-RNA: Targeted RNA
